# Stability analysis of dual solutions for mixed convection and thermal radiation with hybrid nanofluid flow past shrinking/stretching curved surface

**DOI:** 10.1038/s41598-023-48728-8

**Published:** 2023-12-07

**Authors:** Wubshet Ibrahim, Tezera Gizewu

**Affiliations:** Department of Mathematics, Ambo University, Ambo, Ethiopia

**Keywords:** Mathematics and computing, Nanoscience and technology

## Abstract

This study portrays the stability analysis and dual solutions of mixed convection and thermal radiation of hybrid nano-fluid flow past stretching/shrinking a curved surface in the presence of injection/suction conditions. A hybrid nano-fluid, in which water is used as the base fluid, copper and alumina are used as nano-particles, and the magnetic field is taken into account. The present study’s findings will provide fruitful implications for future research in the field of fluid dynamics. The bvp4c method using Matlab software is implemented to get the numerical solution of the nonlinear partial differential equation transformed into the ordinary differential equation. The behavior of the first and second solutions under the governing parameters on the curved surface of dimensionless velocity $g'(\xi )$, shear stress profile $g''(\xi )$, temperature profile $\theta (\xi )$, skin friction coefficient *Cfs*, and local Nusselt’s number *Nus* were visualized in figurative and tabular form. From this the following are investigated: as the values of $\phi j$ increase, the velocity profile for the second solution decreases, and the opposite trend is observed for the first solution. For the values of *K* and $\lambda 1$, the shear stress profile increased for the first solution, and the opposite trend was observed for the second solution, though after some interval points, the inverse of this statement was observed.For the values of $S,Pr,Rd,M, \;\text{and}\; \lambda$, the upwind thermal boundary layer of the first solution is larger than the second solution.For the value of *M* uphill, the estimation of the absolute value of $\lambda ci$ increases for both the skin friction coefficient and the local Nusselt number. In the second solution, increasing the values of $\beta$, *Pr*, *S*, *Ec*, and *M* has a similar effect on $g''(0)$, *Cfs*, -$\theta '(0)$, and *Nus*. In the first solution, increasing the values of *Ec*, *S*, and *Pr* on $g''(0)$ and *Cfs* results in a decrease. The first solution has a positive eigenvalue, whereas the second solution has a negative eigenvalue. Agreement between the present analysis and literature is acceptable.

## Introduction

Nowadays, many researchers are paying attention to the examination of hybrid nano-fluid flow past a shrinking/stretching surface due to several implications in both industrial and manufacturing processes. These include polymer and wire drawing, hot rolling, rubber sheets, the performance of lubricants and paints, the aerodynamic extrusion of plastic sheets, metal spinning, and polymer extrusion. Hence, Yashkun et al.^1^ evaluated the characteristics of thermal radiation and suction influences on linear shrinking and stretching a sheet under magnetohydrodynamic hybrid nano-fluid for the existence of dual solutions. Waini et al.^2^ also discussed the thermal radiation effect on a vertically shrinking sheet with mixed convection magnetohydrodynamic flow in the presence of dust particles and ${{CuAl}}_{2} {{O}}_{3}$ nanoparticles. The stretching sheet in the presence of a heat sink/source, thermal radiation, and chemical reactions of first-order non-Newtonian nanofluid mixed convection flow was addressed by Hayat et al.^3^. Furthermore, Jamaludin et al.^4^ reported a numerical investigation of the vertical shrinking/stretching of a sheet under the influence of a heat source/sink, suction, and thermal radiation at the stagnation point in the presence of mixed convection flow and water as the base fluid, and with nanoparticles as titania $TiO_{2}$, copper Cu, and alumina Al_2_O_3_.

Searching for a new nanoparticle that enhances the heat transfer rate with minimum cost is the duty of noway researchers, but it is a challenging task for scientists in the field of fluid dynamics. The suspension of one type of nanoparticles into a nanofluid containing nanoparticles of other metals or non-metals can enhance the thermal properties to obtain a new kind of nanofluid, which is called hybrid nanofluid. In different industrial areas like nuclear system cooling, microelectronics, naval structures, manufacturing, biomedicine, and drug reduction, hybrid nanofluids have many applications. Yashkun et al.^5^ used water as the base fluid and copper and aluminum oxide as the nanoparticles, with Joule heating and mixed convection under an exponentially shrinking/stretching surface in the presence of heat transfer. A numerical investigation of unsteady mixed convection, magnetohydrodynamic flow of heat transfer, and hybrid nanofluid pasts of permeable vertical plates is reported by Wahid et al.^6^. Muhammad et al.^7^ computed the combined effects of mixed convection, slip, and dissipation due to the heating of hybrid nanofulids formed from Cu and MWCNTs past a surface with a curved-shaped body. Further, many researchers also investigated the effects of hybrid nanofluid flow^8–10^.

In varying practical applications such as transportation, electronics, etc., that is, a modern cutting-edge adhesive technologies, such as a flow past stretching a curved sheet have a wide range of applications. Some of the applications are growing crystals structures, plastic sheets preparation, manufacturing of electronic chips and materials, paper industry, cooling process. Due to this case, the curved sheet connected to the flow has come to the attention of more researchers in the present day. Researchers such as Rosca and Pop^11^ have reported on an electrical conduction fluid of incompressible, viscous, laminar, two-dimensional, unsteady flow with past shrinking sheets in the presence of a transversely identical magnetic field under a curved surface. Also, Revathi et al.^12^ investigated the influence of CH3OH + $SiO_{2}$ + $Al_{2}O_{3}$, a methanol based hybrid nano-fluid, on a curved stretching surface in the presence of thermal radiation and cross diffusion due to increased activation energy and Brinkman number. The exception to the great output devices of entropy generation, activation energy due to forced natural convection flow over a curved surface of fully developed Darcy-Forchheimer is analyzed by Muhammad et al.^13^. Ahmed et al.^14^ developed the phenomena of heat and mass transport under a curving, expanding sheet of Cu-CuO dependent on sodium alginate, NaAlg hybrid nanofluid with boundary layer flow in the presence of nonlinear thermal radiation, chemical reactions, and a magnetic field. Ijaz et al.^15^ discussed the behavior of the entropy optimization rate of magnetohydrodynamic flow due to a curved stretching surface in the presence of Joule heating, heat generation, and viscous dissipation. Ibrahim and Gizewu^16^ reported the characteristic magnetohydrodynamic flow of a third-order slip boundary condition for a curved stretching sheet under gyrotactic microorganisms and bio-convective entropy generation in the presence of Dufour and Soret effects.

The phenomenon in which energy spreads from a heated surface to its absorption point in all directions in the form of electromagnetic waves is called thermal radiation. The thermal agitation of composite molecules in the body generates thermal radiation. Heating of the room due to an open fireplace is a common example of thermal radiation.The sun, the light bulb, and microwave radiation are typical examples. Furthermore, technological applications of it can be seen in solar power, nuclear power plants, combustion chambers, and chemical processes. The amount of heat energy emitted by the heat surface per unit area is directly proportional to the fourth power of the surface’s absolute temperature, as stated by Stefan Boltzmann’s law of radiative heat transfer. Wahid et al.^17^ evaluated the impacts of radiative mixed convection flow with a hybrid nano-fluid, copper-alumina/water, due to the existence of a magnetic field. Bejawada and Nandeppanavar^18^ analyzed the influence of thermal radiation in the presence of micro-polar fluid with magnetohydrodynamic heat transfer flow past a moving porous vertical plate. Lv et al.^19^ explored the behavior of activation energy and chemical reactions with two horizontal infinite plates without nano-fluid flow in the presence of variable thermal conductivity, the Hall effect, and thermal radiation past a permeable and stretchable sheet. Hussain et al.^20^ analyzed the impact of boundary layer flow on velocity slip, thermal radiation at heat absorption, and magnetohydrodynamics past a permeable exponentially stretching surface. The impact of magnetohydrodynamics, chemical reactions, and thermal radiation, in the presence of melting heat transfer, Brownian motion, and thermophoresis effects is due to a non-linear stretching surface, as discussed by Krishnamurthy et al.^21^. For more information, see^22^.

Mixed convection is a blend of free and forced convection in the fluid flow. When the effects of forced flow and free convection/buoyant forces in forced convection are present, a considerable mixed convection flow is produced. Jamaludin et al.^23^ presented a theoretical model for hybrid nano-fluids with mixed convection boundary layer flow due to an exponentially shrinking/stretching surface in the presence of injection/suction and viscous dissipation under the porous medium. Gohar et al.^24^ explored the influence of mixed convection flow in Casson hybrid nanofluid under a curved past stretching sheet on the contribution, according to Darcy-Forchheimer, of permeable media in an incompressible viscous fluid flow. Ibrahim and Gizewu^25–27^ examine mixed convection flow with modified Fick’s and Fourier’s diffusion theories of non-Newtonian tangent hyperbolic fluid in the presence of convective and slip boundary conditions past the non-uniform thickness, bi-directional stretching sheet and entropy generation under a thin film flow boundary condition.

Injection or Suction of fluid through the bounding surfaces, as a consequence, affect the rate of heat transfer from the bounding surfaces, as, for example, in mass transfer cooling, it can significantly change the flow field. In general, injection acts in the opposite manners whereas suction tends to increase the skin-friction and heat transfer coefficients . Injection/withdrawal of fluid through porous cooled or heated surface is of general interest in practical problems involving control of boundary layers, film cooling,etc. This can lead to enhanced cooling /heating of the system and can help from laminar flow to delay the transition^28–30^.

Existence of dual solutions in numerical computing have becoming an important topic in fluid dynamics problems for researchers to study its physical significant. Some of the researchers’ previous works on dual solutions are:- Shi et al.^31^ reported the impacts of the dual solution on Maxwell-fluid flow with mixed convection and thermal radiation in the presence of thermophoresis and Brownian motion past an exponentially shrinking sheet. Yahaya et al.^32^ evaluated the characteristics of a dual solution of two-dimensional unsteady mixed convection and thermal radiation flow for a Riga-plate with a stagnation point over convective boundary conditions. Nadeem et al.^33^ investigated the behavior of micro-polar hybrid nano-fluids with boundary layer flow past a shrinking/stretching surface by using the Runge–Kutta Butcher method along with the Nachtsheim–Swigert iteration with the existence of dual solutions. Naramgari et al.^34^ examined the characteristics of dual solutions in magnetohydrodynamics and mixed convection buoyancy of a non-isothermal stretching sheet in the presence of radiation, injection/suction, chemical reactions, a heat sink/source, and a magnetic field. Mousavi et al.^35^ and De et al.^36^ discuss the influence of a dual solution on the magnetic hydrodynamics of MgO–Ag/water hybrid nano-fluids of Casson flow past a shrinking/stretching surface in the presence of radiation, suction, and convective boundary conditions.

The aim of this article is to investigate the stability analysis and dual solutions of mixed convection with thermal radiation flow of *Cu*–*Al*$_{2}O_{3}/H_{2}O$ hybrid nano-fluid over a curved stretching/shrinking surface due to convective and slip conditions in the presence of suction/injection and magnetic field effects. The findings of the present article will provide fruitful results in the future research in the field of fluid dynamics. To the best of the author’s knowledge, these investigation have not been reported by any researcher yet. This article fills this huge gap in the existing literature.

## Problem formulation

**Figure 1:**
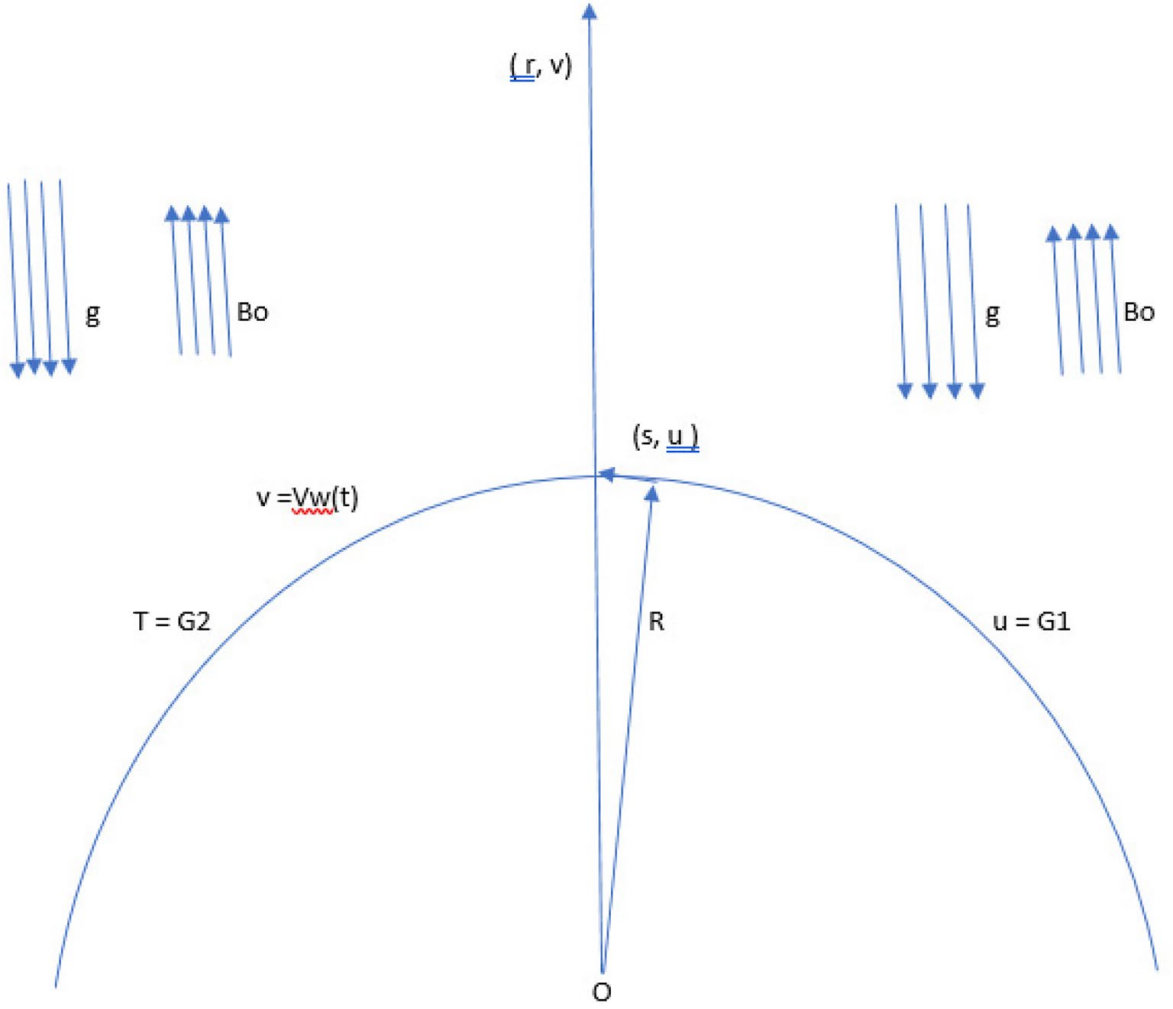
Diagram of the present work.

In this article, 2D, incompressible, viscous boundary layer flow of hybrid nanofluid with the stability analysis for the existence of dual solutions of mixed convection with thermal radiation flow of *Cu*–*Al*${_{2}} {{O}}_{3} {{/H}}_{2} {{O}}$ hybrid nano-fluid over a curved stretching/shrinking surface due to convection and slip conditions in the presence of suction/injection are considered. *r* and *s* are the coordinates of a radius of curvature *R*. (*u*, *v*) are the velocity components along the *s* and *r* directions, and *a* is the positive constant, where *g* is the acceleration due to gravity. The magnetic field *Bo* is taken into account in determining the direction of the flow. A hybrid nano-fluid, in which $H_{2}O$ is used as the base fluid and *Cu* and $Al_{2}O_{3}$ are used as solid nanoparticles, is taken into consideration. Figure 1 presents the physical flow model of the present paper. A system of differential equations under the above assumption for the boundary layers of the flow problem in unsteady form are govern as (see^5,7,11,12,16^):

Equation of continuity:

1 $$\begin{aligned}{} & {} \frac{\partial \Pi v}{\partial r} + R\frac{\partial u}{\partial s}=0, \end{aligned}$$

Equations of momentum:

2 $$\begin{aligned}{} & {} \frac{\partial P}{\partial r} = \frac{\rho _{hnf} u^{2}}{\Pi }, \end{aligned}$$

3 $$\begin{aligned}{} & {} \frac{\partial u}{\partial t} + \frac{R u}{\Pi } \frac{\partial u}{\partial s} + v\frac{\partial u}{\partial r} + \frac{ u v}{\Pi } =\frac{\mu _{hnf}}{\rho _{hnf}}\bigg (\frac{\partial ^{2} u}{\partial r^{2}} + \frac{1}{\Pi } \frac{\partial u}{\partial r} - \frac{u }{(\Pi )^{2}}\bigg ) - \frac{\sigma _{hnf} Bo^{2}}{\rho _{hnf}}u - \frac{ R }{\rho _{hnf}(\Pi )}\frac{\partial P}{\partial s} + \frac{g(\beta \rho )_{hnf}}{\rho _{hnf}}(T-T_{\infty }), \end{aligned}$$

Equation of energy:

4 $$\frac{{\partial T}}{{\partial t}} + v\frac{{\partial T}}{{\partial r}} + \frac{{Ru}}{\Pi }\frac{{\partial T}}{{\partial s}} = \frac{{K_{{hnf}} }}{{(\rho Cp)_{{hnf}} }}\left( {\frac{1}{\Pi }\frac{{\partial T}}{{\partial r}} + \frac{{\partial ^{2} T}}{{\partial r^{2} }}} \right) + \frac{{\sigma _{{hnf}} Bo^{2} u^{2} }}{{(\rho Cp)_{{hnf}} }} + 16\frac{{\sigma ^{*} T_{\infty }^{3} }}{{3k^{*} }}\left( {\frac{1}{\Pi }\frac{{\partial T}}{{\partial r}} + \frac{{\partial ^{2} T}}{{\partial r^{2} }}} \right),$$

where $$\Pi = r+R$$

The boundary conditions defined by^11,16^ are as the follows:

5 $$\begin{aligned}{} & {} T = G2 = T_{w} + K_{hnf}{\lambda _{3}}\frac{\partial T}{\partial r},\quad u = G1 = U_{w}\lambda + \mu _{hnf} {\lambda _{2}}\frac{\partial u}{\partial r}, \quad v= V_{w}, \text { at} \quad r=0, \end{aligned}$$

6 $$\begin{aligned}{} & {} T \rightarrow T_{\infty }, u \rightarrow 0, \frac{\partial u}{\partial r} \rightarrow 0, \quad \text {for r} \rightarrow \infty . \end{aligned}$$

$V_{w} = -\sqrt{a\nu _{f}}S$, $U_{w} = as$ and $\lambda>0, \lambda< 0, S > 0, S<0, {\lambda _{2}}$ and ${\lambda _{3}}$ are representing stretching sheet, shrinking sheet, suction, injection, velocity slip and thermal jump parameters respectively.

## Steady-state case ($\frac{\partial u}{\partial t} = 0$)

The subsequent similarity variables employed in the assumed steady-state case of the governing problem in Eqs. (1)–(6) given by^11^ and^16^ as:

7 $$\begin{aligned}{} & {} \xi = \sqrt{\frac{a}{\nu _{f}}}r, \quad u = asg'(\xi ), \quad v = \frac{R}{\Pi }\sqrt{a\nu _{f}} g(\xi ), \theta (\xi ) = \frac{T-T_{\infty }}{T_{w}-T_{\infty }},\quad p = \rho _{f} a^2s^2P(\xi ), \end{aligned}$$

When the similarity transformation [see Eq. (19)] is adopted into Eq. (1) it is sufficed while the steady form of Eqs.(2)–(4) are altered into

8 $$\begin{aligned}{} & {} \rho _{hnf}\frac{g'^2}{K+\xi } = \frac{\partial P}{\partial \xi }, \end{aligned}$$

9 $$\begin{aligned}{} & {} \frac{\mu _{f}}{\mu _{hnf}} \frac{K}{K+\xi }P = g''' + \frac{g''}{K + \xi } - \frac{g'}{(K+\xi )^2}+ \frac{\mu _{f}}{\mu _{hnf}}\rho _{hnf}(-\beta (g' + \frac{\xi }{2})+\frac{K}{(K+\xi )^2}gg'-\nonumber \\{} & {} \frac{K}{K+\xi }g'^2 + \frac{K}{K+\xi }gg'') - \frac{\mu _{f}}{\mu _{hnf}} \sigma _{hnf} Mg'+ \frac{\mu _{f}}{\mu _{hnf}}(\beta \rho )_{hnf}\lambda 1\theta , \end{aligned}$$

10 $$\begin{aligned}{} & {} (Rd + \frac{k_{hnf}}{k_{f}})(\frac{1}{K+\xi }\theta ' + \theta '')- Pr\frac{(\rho Cp)_{hnf}}{(\rho Cp)_{f}}(\frac{\xi \beta \theta '}{2} - \frac{K}{K+\xi }g\theta ') + \frac{\sigma _{hnf}}{\sigma _{f}}EcMg'^2 = 0. \end{aligned}$$

where $$Pr = \frac{\nu _{f}}{\alpha _{f}}$$, $$\beta =\frac{\alpha }{a}$$, $$K=R\sqrt{\frac{a}{\nu _{f}}}$$, $$\lambda 1 = \frac{Gr}{(Res)^2}$$, $$Gr =\frac{g\beta _{f}(T_{w}-T_{\infty })s^3}{\nu ^{2}_{f}}$$,

$Rd = \frac{16\sigma ^* T^{3}_{\infty }}{3K*K_{f}}$, $Ec = \frac{\mu _{f} a^2s^2}{K_{f}(T_{w}-T_{\infty })}$, $M = \frac{\sigma Bo^{2}}{a\rho }$ are Prandtl number, parameter of unsteadiness, curvature parameter, mixed convection parameter, local Grashof number, radiation parameter, Eckert number and Hartman number parameter respectively.

When we compare Eqs. (9) and (8), we obtain:

11 $$\begin{aligned} & g^{{iv}} + \frac{2}{{K + \xi }}g^{\prime\prime\prime} - \frac{1}{{(K + \xi )^{2} }}g^{\prime\prime} + \frac{1}{{(K + \xi )^{3} }}g^{\prime} + \frac{{\mu _{f} }}{{\mu _{{hnf}} }}\rho _{{hnf}} \left[ {\frac{K}{{K + \xi }}(gg^{\prime\prime\prime} - g^{\prime}g^{\prime\prime}) + \frac{K}{{(K + \xi )^{2} }}(gg^{\prime\prime} - (g^{\prime})^{2} )} \right. \\ & \quad \left. { - \frac{K}{{(K + \xi )^{3} }}gg^{\prime} - \frac{\beta }{{(K + \xi )}}(g^{\prime} + \frac{\xi }{2}g^{\prime\prime}) - \frac{\beta }{2}(3g^{\prime\prime} + \xi g^{\prime\prime\prime})} \right] - \frac{{\mu _{f} }}{{\mu _{{hnf}} }}\sigma _{{hnf}} M(g^{\prime\prime} + \frac{1}{{K + \xi }}g^{\prime}) \\ & \quad + \frac{{\mu _{f} }}{{\mu _{{hnf}} }}(\beta \rho )_{{hnf}} \lambda 1\left( {\theta ^{\prime} + \frac{\theta }{{K + \xi }}} \right) = 0 \\ \end{aligned}$$

12 $$\begin{aligned}{} & {} (Rd + \frac{k_{hnf}}{k_{f}})\bigg (\frac{1}{K+\xi }\theta ' + \theta ''\bigg )- Pr\frac{(\rho Cp)_{hnf}}{(\rho Cp)_{f}}\bigg (\frac{\xi \beta \theta '}{2} - \frac{K}{K+\xi }g\theta '\bigg ) + \frac{\sigma _{hnf}}{\sigma _{f}}EcMg'^2 = 0 \end{aligned}$$

The dimensionless forms of the associated boundary conditions are:

13 $$\begin{aligned}{} & {} g(0) = S, g'(0) = \lambda + \frac{\mu _{hnf}}{\mu _{f}}\lambda 2 g''(0), \theta (0)= \frac{K_{hnf}}{K_{f}}\lambda 3 \theta '(0) + 1,\nonumber \\{} & {} g'(\xi )\rightarrow 0, g''(\xi )\rightarrow 0, \theta (\xi )\rightarrow 0 \quad \text { as} \quad \xi \rightarrow \infty . \end{aligned}$$

where

$$\begin{aligned} \lambda 2 = S_{1}\sqrt{a\mu _{f}\rho _{f}}, \lambda 3 = S_{2}\sqrt{\frac{a}{\nu _{f}}K_{f}} \end{aligned}$$

From Eq. (9) *P* would be written as the following:

14 $$\begin{aligned} P & = \frac{1}{{\frac{{\mu _{f} }}{{\mu _{{hnf}} }}\frac{K}{{K + \xi }}}}[g^{\prime\prime\prime} + \frac{{g^{\prime\prime}}}{{K + \xi }} - \frac{{g^{\prime}}}{{(K + \xi )^{2} }} + \frac{{\mu _{f} }}{{\mu _{{hnf}} }}\rho _{{hnf}} ( - \beta (g^{\prime} + \frac{\xi }{2}) + \frac{K}{{(K + \xi )^{2} }}gg^{\prime} \\ & \quad - \frac{K}{{K + \xi }}g^{\prime2} + \frac{K}{{K + \xi }}gg^{\prime\prime}) - \frac{{\mu _{f} }}{{\mu _{{hnf}} }}\sigma _{{hnf}} Mg^{\prime} + \frac{{\mu _{f} }}{{\mu _{{hnf}} }}(\beta \rho )_{{hnf}} \lambda 1\theta ], \\ \end{aligned}$$

The skin friction coefficient $Cf_{r}$ and local Nusselt number $Nu_{l}$ (see^37^) are the physical interests of engineering quantities expressed as follows:

15 $$\begin{aligned} \frac{\tau _{w}}{(as)^{2}\rho _{f}} = Cf_{r}, \frac{q_{w}s}{(T_{w}-T_{\infty })k_{f}} = Nu_{l} \end{aligned}$$

where

16 $$\begin{aligned} \tau _{w} = (\frac{\partial u}{\partial r}-\frac{u}{\Pi })\mu _{hnf}, q_{w} = -k_{hnf}\frac{\partial T}{\partial r} at r= 0 \end{aligned}$$

after implementing Eqs. (19), (15) and (16),

17 $$\begin{aligned} Cfs = (g''(0)\bigg (1-\frac{\lambda 2}{K}\frac{\mu _{hnf}}{\mu _{f}}\bigg )- \frac{\lambda }{K} )\frac{\mu _{hnf}}{\mu _{f}}, Nus = -\bigg (\frac{K_{hnf}}{K_{f}} + Rd\bigg )\theta '(0) \end{aligned}$$

where

$$\begin{aligned} Cfs = (Re_{s})^{\frac{1}{2}} Cf_{r}, Nus = (Re_{s})^{\frac{-1}{2}} Nu_{l}, Re_{s} = \frac{as^{2}}{\nu _{f}} \end{aligned}$$

are represented by the reduced local Nusselt’s number, reduced skin friction coefficient, and local Reynolds number, respectively.

The thermo-physical characteristics of nano-particles and base fluid as given by^5^ in the following Table 1:

**Table 1:** Thermophysical characteristics of nanoparticles and base fluid.

Nano particles and basefluids	Thermophysical characteristics				
	$\rho \;{(kg/m^3)}$	*Cp* (*J*/*kgK*)	k (W/mK)	$\beta *10^{-5}(1/K)$	$\sigma {(S/m)}$
$Al_{2}O_{3}$	3970	765	40	0.85	$3.69*10^7$
Cu	8933	385	400	1.67	$5.96 *10^7$
$H_{2}O$	997.1	4179	0.613	21	0.05

The hybrid nano-fluid under consideration are given by^5^:

18 $$\begin{aligned} (\rho Cp)_{hnf}&= 1-\phi j[(1-\phi i)(\rho Cp)_{f} + \phi i(\rho Cp) ni]+ \phi j(\rho Cp) nj, \nonumber \\ \sigma _{hnf}&= \frac{\sigma nj + 2\sigma nf - 2\phi j(\sigma nf - \sigma nj)}{\sigma nj + 2\sigma nf + \phi j(\sigma nf - \sigma nj)}*\sigma nf \nonumber \\ \beta _{hnf}&= 1-\phi j[(1-\phi i)\beta _{f} + \phi i\beta n i]+ \phi j\beta n j, \nonumber \\ \rho _{hnf}&= 1-\phi j[(1-\phi i)\rho _{f} + \phi i\rho ni]+ \phi j\rho nj, \nonumber \\ k_{nf}&= \frac{kni + 2kf - 2\phi i(kf - kni)}{kni + 2kf + \phi j(kf - kni)}*kf, \nonumber \\ k_{hnf}&= \frac{kn j + 2knf - 2\phi j(knf - knj)}{knj + 2knf + \phi j(knf - kn j)}*knf, \nonumber \\ \sigma _{nf}&= \frac{\sigma ni + 2\sigma f - 2\phi i(\sigma f - \sigma ni)}{\sigma ni + 2\sigma f + \phi j(\sigma f - \sigma ni)}*\sigma f, \nonumber \\ \mu _{hnf}&= \frac{\mu _{f}}{(1-\phi i)^{\frac{5}{2}}(1-\phi j)^{\frac{5}{2}}}. \end{aligned}$$

## Stability analysis

Researchers^38,39^ were reported the existence of dual solutions. To determine these solutions are realizable physically, stability analysis of equations (11)-(13) is required. In order to observe which solution is realizable the parameter $\tau$ is utilized with an initial value problem where new variables given as the following:

19 $$\begin{aligned} \xi = \sqrt{\frac{a}{\nu _{f}}}r, \quad u = as\frac{\partial g}{\partial \xi }(\xi ,\tau ), \quad v = \frac{R}{\Pi }\sqrt{a\nu _{f}} g(\xi ,\tau ), \theta (\xi ,\tau ) = \frac{T-T_{\infty }}{T_{w}-T_{\infty }},\quad p = \rho _{f} a^2s^2P(\xi ,\tau ),\quad \tau =at \end{aligned}$$

One obtains after substitution of (19) into the unsteady form of (1) and (6) are:

20 $$\begin{aligned} & \frac{{\partial ^{4} g}}{{\partial \xi ^{4} }} + \frac{2}{{K + \xi }}\frac{{\partial ^{3} g}}{{\partial \xi ^{3} }} - \frac{1}{{(K + \xi )^{2} }}\frac{{\partial ^{2} g}}{{\partial \xi ^{2} }} + \frac{1}{{(K + \xi )^{3} }}\frac{{\partial g}}{{\partial \xi }} + \frac{{\mu _{f} }}{{\mu _{{hnf}} }}\rho _{{hnf}} \left[ {\frac{K}{{K + \xi }}\left( {g\frac{{\partial ^{3} g}}{{\partial \xi ^{3} }} - \frac{{\partial g}}{{\partial \xi }}\frac{{\partial ^{2} g}}{{\partial \xi ^{2} }}} \right)} \right. \\ & \quad \left. { + \frac{K}{{(K + \xi )^{2} }}\left( {g\frac{{\partial ^{2} g}}{{\partial \xi ^{2} }} - \left( {\frac{{\partial g}}{{\partial \xi }}} \right)^{2} } \right) - \frac{K}{{(K + \xi )^{3} }}g\frac{{\partial g}}{{\partial \xi }} - \frac{\beta }{{(K + \xi )}}\left( {\frac{{\partial g}}{{\partial \xi }} + \frac{\xi }{2}\frac{{\partial ^{2} g}}{{\partial \xi ^{2} }}} \right) - \frac{\beta }{2}\left( {3\frac{{\partial ^{2} g}}{{\partial \xi ^{2} }} + \xi \frac{{\partial ^{3} g}}{{\partial \xi ^{3} }}} \right)} \right] \\ & \quad - \frac{{\mu _{f} }}{{\mu _{{hnf}} }}\sigma _{{hnf}} M\left( {\frac{{\partial ^{2} g}}{{\partial \xi ^{2} }} + \frac{1}{{K + \xi }}\frac{{\partial g}}{{\partial \xi }}} \right) + \frac{{\mu _{f} }}{{\mu _{{hnf}} }}(\beta \rho )_{{hnf}} \lambda 1\left( {\frac{{\partial \theta }}{{\partial \xi }} + \frac{\theta }{{K + \xi }}} \right) - \frac{1}{{(K + \xi )}}\frac{{\partial ^{2} g}}{{\partial \xi \partial \tau }} - \frac{{\partial ^{3} g}}{{\partial \xi ^{2} \partial \tau }} = 0, \\ \end{aligned}$$

21 $$\begin{aligned}{} & {} (Rd + \frac{k_{hnf}}{k_{f}})\bigg (\frac{1}{K+\xi }\frac{\partial \theta }{\partial \xi } + \frac{\partial ^{2} \theta }{\partial \xi ^{2}}\bigg )- Pr\frac{(\rho Cp)_{hnf}}{(\rho Cp)_{f}}\bigg (\frac{\xi \beta }{2}\frac{\partial \theta }{\partial \xi } - \frac{K}{K+\xi }g\frac{\partial \theta }{\partial \xi }\bigg ) + \frac{\sigma _{hnf}}{\sigma _{f}}EcM(\frac{\partial g }{\partial \xi })^2 - \frac{\partial \theta }{\partial \tau } = 0, \end{aligned}$$

subject to:

22 $$\begin{aligned}{} & {} g(0,\tau ) = S, \frac{\partial g }{\partial \xi }(0,\tau ) = \lambda + \frac{\mu _{hnf}}{\mu _{f}}\lambda 2 \frac{\partial ^{2} g}{\partial \xi ^{2}}(0,\tau ), \theta (0,\tau )= \frac{K_{hnf}}{K_{f}}\lambda 3 \frac{\partial \theta }{\partial \xi }(0,\tau ) + 1, \quad \text { at} \quad \xi = 0,\nonumber \\{} & {} \frac{\partial g }{\partial \xi }(\xi ,\tau )\rightarrow 0, \frac{\partial ^{2} g}{\partial \xi ^{2}}(\xi ,\tau )\rightarrow 0, \theta (\xi ,\tau )\rightarrow 0 \quad \text { as} \quad \xi \rightarrow \infty . \end{aligned}$$

Then, consider the following perturbation functions which is used to check the stability of the steady flow solutions $g(\xi ) = g_{0}(\xi )$ and $\theta (\xi ) = \theta _{0}(\xi )$ satisfying the boundary value problem (11)–(13) (see^38,39^)

23 $$\begin{aligned} g(\xi ,\tau ) = g_{0}(\xi ) + e^{-\gamma \tau }G(\xi ,\tau ), \quad \theta (\xi ,\tau ) = \theta _{0}(\xi ) + e^{-\gamma \tau }\theta _{1}(\xi ,\tau ). \end{aligned}$$

where unknown eigenvalue parameter is $\gamma$ and the functions $G(\xi ,\tau )$ and $\theta _{1}(\xi ,\tau )$ are relatively small compared to $g_{0}(\xi )$ and $\theta _{0}(\xi )$ respectively. The stability of the solutions are determined by the sign (positive or negative) of the eigenvalues. Putting of (23) into (20)–(22) gives the following linearized eigenvalue problem:

24 $$\begin{aligned}{} & {} \frac{\partial ^{4} G}{\partial \xi ^{4}} + \frac{2}{K+\xi }\frac{\partial ^{3}G }{\partial \xi ^{3}} - \frac{1}{(K+ \xi )^{2}}\frac{\partial ^{2}G }{\partial \xi ^{2}} + \frac{1}{(K+ \xi )^{3}}\frac{\partial G }{\partial \xi } + \frac{\mu _{f}}{\mu _{hnf}}\rho _{hnf}[ \frac{K}{K+ \xi }(g_{0}\frac{\partial ^{3} G }{\partial \xi ^{3}}+ G g'''_{0} - g'_{0}\frac{\partial ^{2} G}{\partial \xi ^{2}} - \nonumber \\{} & {} \frac{\partial G}{\partial \xi }g''_{0})+ \frac{K}{(K+ \xi )^{2}}(g_{0}\frac{\partial ^{2} G}{\partial \xi ^{2}} + G g''_{0} - 2 g'_{0}\frac{\partial G}{\partial \xi })- \frac{K}{(K+ \xi )^{3}}(g_{0}\frac{\partial G}{\partial \xi } + G g'_{0})- \frac{\beta }{(K+ \xi )}(\frac{\partial G }{\partial \xi }+\frac{\xi }{2}\frac{\partial ^{2} G}{\partial \xi ^{2}}) - \nonumber \\{} & {} \frac{\beta }{2}(3\frac{\partial ^{2}G }{\partial \xi ^{2}}+ \xi \frac{\partial ^{3}G }{\partial \xi ^{3}})] - \frac{\mu _{f}}{\mu _{hnf}} \sigma _{hnf} M(\frac{\partial ^{2} G}{\partial \xi ^{2}} +\frac{1}{K + \xi } \frac{\partial G }{\partial \xi }) + \frac{\mu _{f}}{\mu _{hnf}}(\beta \rho )_{hnf} \lambda 1 (\frac{\partial \theta _{1}}{\partial \xi } + \frac{1}{K+ \xi }\theta _{1})+ \nonumber \\{} & {} \frac{\mu _{f}}{\mu _{hnf}}\rho _{hnf}(\frac{1}{(K + \xi )}( \tau \frac{\partial G}{\partial \xi }- \frac{\partial ^{2} G}{\partial \xi \partial \tau }) + \tau \frac{\partial ^{2} G}{\partial \xi ^{2}}- \frac{\partial ^{3} G}{\partial \xi ^{2} \partial \tau })= 0, \end{aligned}$$

25 $$\begin{aligned}{} & {} (Rd + \frac{k_{hnf}}{k_{f}})\bigg (\frac{1}{K+\xi }\frac{\partial \theta _{1}}{\partial \xi } + \frac{\partial ^{2} \theta _{1} }{\partial \xi ^{2}}\bigg )- Pr\frac{(\rho Cp)_{hnf}}{(\rho Cp)_{f}}\bigg (\frac{\xi \beta }{2}\frac{\partial \theta _{1}}{\partial \xi } - \frac{K}{K+\xi }(g_{0}\frac{\partial \theta _{1}}{\partial \xi }+ G\frac{\partial \theta _{0}}{\partial \xi })\bigg ) + \nonumber \\{} & {} \frac{\sigma _{hnf}}{\sigma _{f}}EcM(2\frac{\partial g_{0}}{\partial \xi }\frac{\partial G }{\partial \xi }) + \tau \theta _{1}- \frac{\partial \theta _{1}}{\partial \tau } = 0, \end{aligned}$$

With their corresponding boundary conditions are:

26 $$\begin{aligned} G(0,\tau ) = 0, \frac{\partial G }{\partial \xi }(0,\tau ) = \frac{\mu _{hnf}}{\mu _{f}}\lambda 2 \frac{\partial ^{2} G}{\partial \xi ^{2}}(0,\tau ), \theta _{1}(0,\tau )= \frac{K_{hnf}}{K_{f}}\lambda 3 \frac{\partial \theta _{1}}{\partial \xi }(0,\tau ), \quad \text { at} \quad \xi = 0,\nonumber \\ \frac{\partial G }{\partial \xi }(\xi ,\tau )\rightarrow 0, \frac{\partial ^{2} G}{\partial \xi ^{2}}(\xi ,\tau )\rightarrow 0, \theta _{1}(\xi ,\tau )\rightarrow 0 \quad \text { as} \quad \xi \rightarrow \infty . \end{aligned}$$

by considering $\tau = 0$ (24)–(26) are reduced as the following:

27 $$\begin{aligned}{} & {} G^{iv} + \frac{2}{K+\xi }G''' - \frac{1}{(K+ \xi )^{2}}G'' + \frac{1}{(K+ \xi )^{3}}G' + \frac{\mu _{f}}{\mu _{hnf}}\rho _{hnf}[\frac{K}{K+ \xi } (g_{0}G'''+ G g'''_{0} - g'_{0}G'' - G'g''_{0})+ \nonumber \\{} & {} \frac{K}{(K + \xi )^{2}}(g_{0}G'' + G g''_{0} - 2 g'_{0}G')- \frac{K}{(K+ \xi )^{3}}(g_{0}G' + G g'_{0})- \frac{\beta }{(K+ \xi )}(G'+\frac{\xi }{2}G'') - \nonumber \\{} & {} \frac{\beta }{2}(3G''+ \xi G''')] - 
\frac{\mu _{f}}{\mu _{hnf}} \sigma _{hnf} M(G'' +\frac{1}{K + \xi } G') + \frac{\mu _{f}}{\mu _{hnf}}(\beta \rho )_{hnf} \lambda 1 (\theta '_{1} + \frac{1}{K+ \xi }\theta _{1})+ \nonumber \\{} & {} \frac{\mu _{f}}{\mu _{hnf}}\rho _{hnf}(\frac{1}{(K + \xi )}\tau ( G' + G'') = 0, \end{aligned}$$

28 $$\begin{aligned}{} & {} \frac{1}{Pr}\frac{(\rho Cp)_{f}}{(\rho Cp)_{hnf}}(Rd + \frac{k_{hnf}}{k_{f}})\bigg (\frac{1}{K+\xi }\theta '_{1} + \theta ''_{1} \bigg )- \frac{\xi \beta }{2} \theta '_{1} + \frac{K}{K+\xi }(g_{0}\theta '_{1}+ G \theta '_{0}) + \nonumber \\{} & {} \frac{\sigma _{hnf}}{\sigma _{f}}EcM(2 g_{0}G') + \tau \theta _{1} = 0, \end{aligned}$$

With their corresponding boundary conditions are:

29 $$\begin{aligned}{} & {} G(\xi ) = 0,\quad G'(\xi ) = \frac{\mu _{hnf}}{\mu _{f}}\lambda 2 G''(\xi ), \quad \theta _{1}(\xi )= \frac{K_{hnf}}{K_{f}}\lambda 3 \theta '_{1}(\xi ), \quad \text { at} \quad \xi = 0,\nonumber \\{} & {} G'(\xi )\rightarrow 0, \quad G''(\xi )\rightarrow 0, \quad \theta _{1}(\xi )\rightarrow 0 \quad \text { as} \quad \xi \rightarrow \infty . \end{aligned}$$

It should be reported that for various values of *Pr* the stability of the steady flow solution $g_{0}(\xi )$ is determined by the smallest eigenvalue $\gamma$. According to^38,39^, the range of possible eigenvalues can be determined by relaxing a boundary condition on $G_{0}(\xi )$. Thus, we relax the condition that $G_{0}''(\xi ) \rightarrow 0$ as $\xi \rightarrow \infty$ and for a fixed value of $\gamma$ (27)–(28) along with the new boundary condition $G_{0}'''(0) = 1$ has to be solved.

## Numerical solution

To find the numerical solution of the non-linear ordinary differential equations subject to the boundary conditions of the flow problem, bvp4c solver using Matlab software was employed. To solve the non-linear ordinary differential equations (11) and (12), with their respective boundary conditions (13), we form a system of first order ordinary differential equations by letting in the form of $g = f1$; $g' = f2$; $g'' = f3$;$g''' = f4$; $g^{iv} = f4'$; $\theta = f5$; $\theta ' = f6$; $\theta '' = f6'$; $gi(a) = fi(a)$, $gi(b) = fi(b)$, $\theta i(a) = fi(a)$, $\theta i(b) = fi(b)$ where $i = 1,2,3,\cdots 6$, $a= 0$ and $b \rightarrow \infty$.

Then Eqs. 11–13 are reduced to the following form:

30 $$\begin{aligned}{} & {} f4'+ \frac{2}{K+\xi }f4- \frac{1}{(K+ \xi )^{2}}f3+ \frac{1}{(K+ \xi )^{3}f2} + \frac{\mu _{f}}{\mu _{hnf}}\rho _{hnf}[\frac{K}{K+ \xi }(f1f4-f2f3)+ \nonumber \\{} & {} \frac{K}{(K+ \xi )^{2}}- \frac{K}{(K+ \xi )^{3}}f1f2- \frac{\beta }{(K+ \xi )}(f2+\frac{\xi }{2}f3) - \frac{\beta }{2}(3f3+ \xi f4)] - \nonumber \\{} & {} \frac{\mu _{f}}{\mu _{hnf}} \sigma _{hnf} ( Mf3 +\frac{1}{K + \xi } Mf2)+ \frac{\mu _{f}}{\mu _{hnf}}(\beta \rho )_{hnf} \lambda 1 (f6 + \frac{f5}{K+ \xi }) = 0, \end{aligned}$$

31 $$\begin{aligned}{} & {} (Rd + \frac{k_{hnf}}{k_{f}})\bigg (\frac{1}{K+\xi }f6 + f6'\bigg )- Pr\frac{(\rho Cp)_{hnf}}{(\rho Cp)_{f}}\bigg (\frac{\xi \beta f6}{2} - \frac{K}{K+\xi }f1f6\bigg ) + \frac{\sigma _{hnf}}{\sigma _{f}}EcMf2^2 = 0, \end{aligned}$$

32 $$\begin{aligned}{} & {} f1(a) = S, f2(a) = \lambda + \frac{\mu _{hnf}}{\mu _{f}}\lambda 2 f3(a), f5(a)= \frac{K_{hnf}}{K_{f}}\lambda 3 f6(a) + 1,\nonumber \\{} & {} f2(b)= 0, f3(b)= 0, f5(b)= 0. \end{aligned}$$

## Outcomes and discussion

The following values were used to analyze the effects of current governing parameters: $M= 0.9, Ec = 1, S = 5, \xi = 0.4, \beta = -1, K= 50,Rd = 0.2, \lambda 1 = 0.1, \lambda 2 = 0.2, \lambda 3 = 0.2, Pr = 6.2, \lambda = -10, \phi i = 0.05$ and $\phi j = 0.02$. The characteristics of stability analysis and dual solutions for the dimensionless parameters of the present study are given as follows: The effect of *M*; *Ec*; $\beta$; *K*; *Rd*; *S*; $\lambda 1$; $\lambda 2$; $\lambda 3$; *Pr*; $\lambda$; $\phi i$ and $\phi j$ on the velocity profile, shear stress profile, temperature profile, skin friction coefficient profile, and local Nusselt number profile are shown in Fig. 2, 3, 4, 5, 6, 7, 8, 9, 10, 11, 12, 13, 14, 15, 16, 17, 18, 19, 20, 21, 22, 23, 24 and 25 and Tables 2, 3 and 4.

**Table 2:** reports the comparison with previous work^11^ and present study for different values of *K* on -*Cfs*.

*K*	Earlier work^11^	Present result
5	1.15076	1.150760
10	1.07172	1.071725
20	1.03501	1.035011
25	–	1.02729
30	1.02315	1.023151
35	−	1.012678

**Table 3:** portrays the effect of the dual solutions, which are the first and second branch solutions of *M*, *Ec*, *S*, *Pr*, and $\beta$ respectively, on $g''(0)$ and *Cfs*.

	First Solution		Second Solution	
	$g''(0)$	*Cfs*	$g''(0)$	*Cfs*
*M*				
0.1	− 12.053993989	− 7.117762911	− 3.966122351	− 2.341955587
0.5	− 14.812652666	− 8.746723273	− 4.281819123	− 2.528371374
0.9	− 18.689227112	− 11.035801718	− 4.594119725	− 2.712781757
*Ec*				
1.4	− 14.115628217	− 8.335137306	− 3.997838860	− 2.360683868
1.5	− 49.953706046	− 29.497163883	− 4.005762982	− 2.365362983
*S*				
3.0	− 14.721418769	− 8.692850569	− 4.650723570	− 2.746205761
3.1	− 17.505810329	− 10.337005941	− 4.856004701	− 2.867422216
3.2	− 17.512479910	− 10.340944262	− 5.102713780	− 3.013101460
*Pr*				
6.5	− 24.922970948	− 14.716765115	− 3.969043382	− 2.343680427
6.6	− 33.947385712	− 20.045591789	− 3.970057192	− 2.344279072
7.9	− 47.552644190	− 28.079360868	− 3.985280345	− 2.353268191
$$\beta$$				
− 1.6	− 62.930481526	− 37.159820036	− 2.503883878	− 1.478518391
− 1.7	− 26.271574666	− 15.513102124	− 2.836181901	− 1.674737051
− 1.8	− 15.472132020	− 9.136139236	− 3.189816616	− 1.883554814

**Table 4:** portrays the effect of the dual solutions, which are the first and second branch solutions of *M*, *Ec*, *S*, *Pr*, and $\beta$ respectively, on -$\theta '(0)$ and *Nus*.

	First Solution		Second Solution	
	$-\theta '(0)$	*Nus*	$-\theta '(0)$	*Nus*
*M*				
0.1	2.219550377	3.890691498	− 10.400157272	− 18.230630806
0.5	2.223300712	3.897265531	− 51.703571400	− 90.632160352
0.9	2.474850950	4.338212662	− 92.515268628	− 162.171750118
*Ec*				
1.4	− 2.581521228	− 4.525196995	− 14.552007521	− 25.508487002
1.5	2.590076063	4.540192925	− 15.589232815	− 27.326658682
*S*				
3.0	1.492252840	2.615798001	− 20.628188767	− 36.159539110
3.1	1.757807956	3.081294545	− 24.148176979	− 42.329792487
3.2	1.511635244	2.649773781	− 28.521206678	− 49.995358290
*Pr*				
6.5	12.045601025	21.114960033	− 11.184958116	− 19.606322929
6.6	1.043393188	1.828983495	− 11.461034380	− 20.090262193
7.9	−	−	− 15.823202856	− 27.736789155
$$\beta$$				
− 1.6	2.364124851	4.144118806	− 9.930666304	− 17.407651281
− 1.7	2.242992137	3.931782999	− 10.042450460	− 17.603599824
− 1.8	−	−	− 10.157721711	− 17.805660963

**Figure 2:**
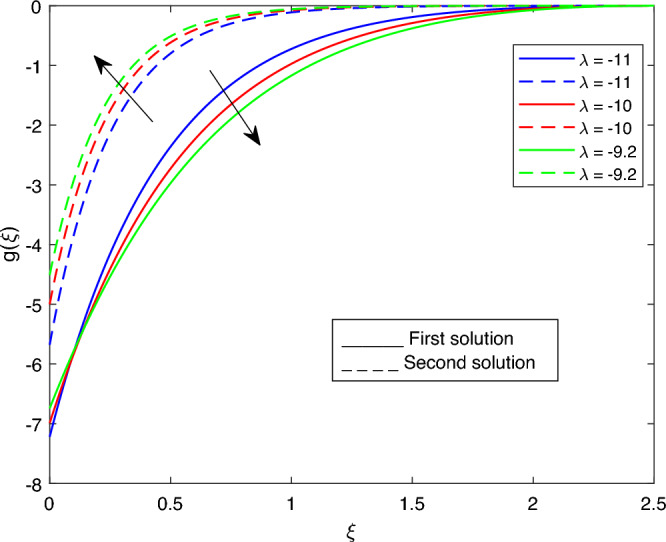
Portrays the existence of dual solutions on $g'(\xi )$ with variation of $\lambda$ when $M= 0.9, Ec = 1, \xi = 0.4, \beta = -1, K= 50,Rd = 0.2, S= 5, \lambda 1 = 0.1, \lambda 2 = 0.2, \lambda 3 = 0.2, Pr = 6.2, \phi i = 0.05$ and $\phi j = 0.02$.

**Figure 3:**
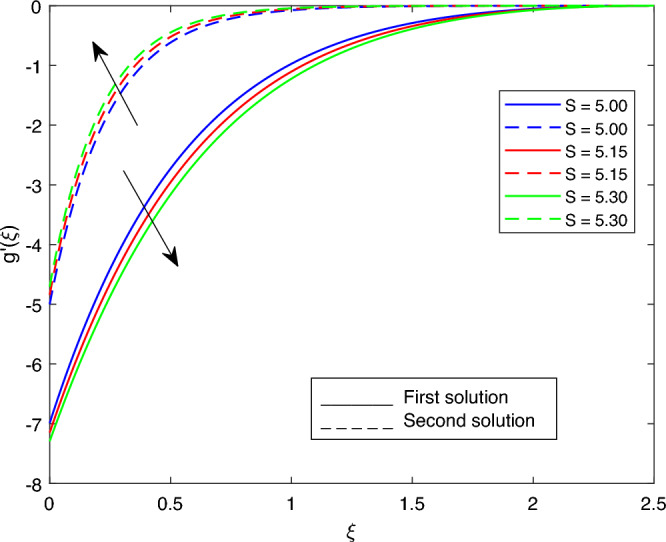
Portrays the existence of dual solutions on $g'(\xi )$ with variation of *S* when $M= 0.9, Ec = 1, \xi = 0.4, \beta = -1, K= 50,Rd = 0.2, \lambda 1 = 0.1, \lambda 2 = 0.2, \lambda 3 = 0.2, Pr = 6.2, \lambda = -10, \phi i = 0.05$ and $\phi j = 0.02$.

**Figure 4:**
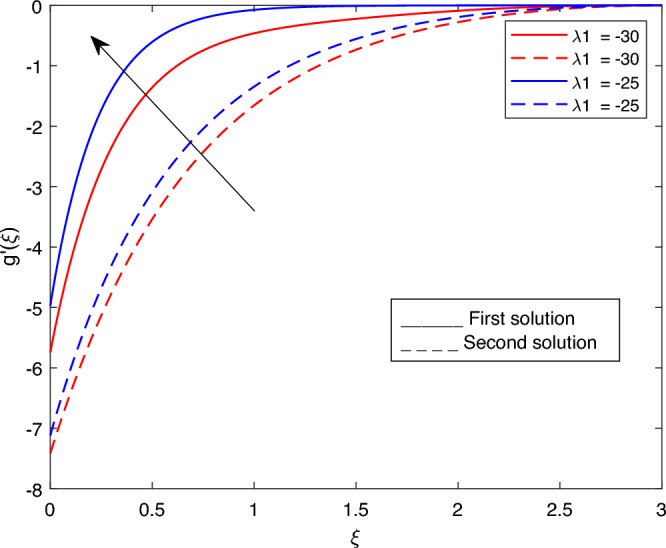
Portrays the existence of dual solutions on $g'(\xi )$ with variation of$\lambda 1$ on $g'(\xi )$ when $M= 0.9, Ec = 1, \xi = 0.4, \beta = -1, K= 50,Rd = 0.2, S= 5, \lambda 2 = 0.2, \lambda 3 = 0.2, Pr = 6.2, \lambda = -10, \phi i = 0.05$ and $\phi j = 0.02$.

**Figure 5:**
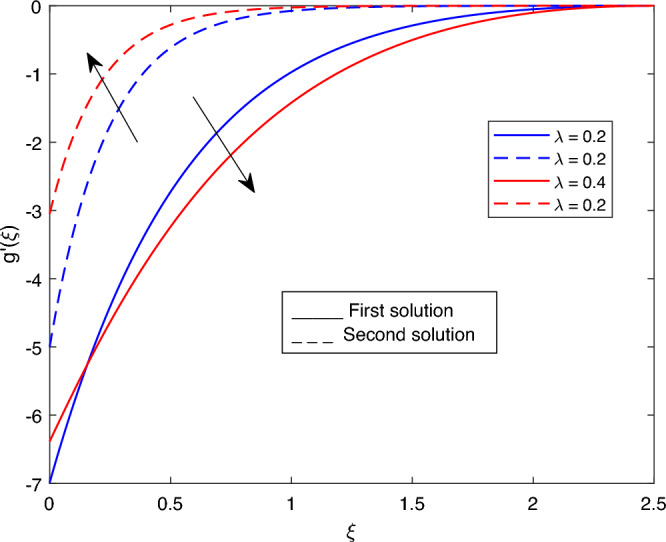
Portrays the existence of dual solutions on $g'(\xi )$ with variation of $\lambda 2$ when $M= 0.9, Ec = 1, \xi = 0.4, \beta = -1, K= 50,Rd = 0.2, S= 5, \lambda 1 = 0.1, \lambda 3 = 0.2, Pr = 6.2, \lambda = -10, \phi i = 0.05$ and $\phi j = 0.02$.

**Figure 6:**
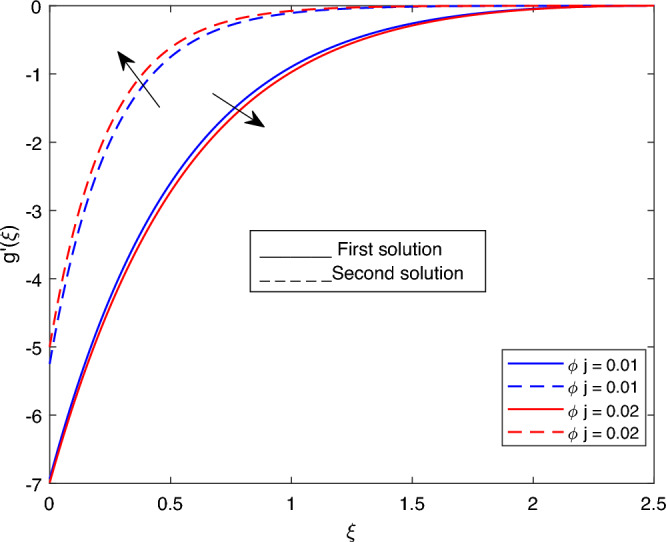
Portrays the existence of dual solutions on $g'(\xi )$ with variation of $\phi j$ when $M= 0.9, Ec = 1, \xi = 0.4, \beta = -1, K= 50,Rd = 0.2, S= 5, \lambda 1 = 0.1, \lambda 3 = 0.2, Pr = 6.2, \lambda = -10, \phi i = 0.05$ and $\phi j = 0.02$.

**Figure 7:**
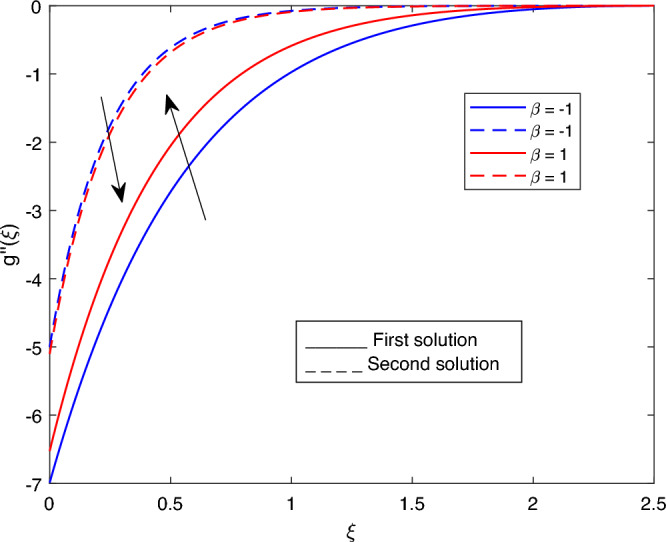
Portrays the existence of dual solutions on $g''(\xi )$ with variation of $\beta$ when $M= 0.9, Ec = 1, \xi = 0.4, \beta = -1, K= 50,Rd = 0.2, S= 5, \lambda 1 = 0.1, \lambda 2 = 0.2, \lambda 3 = 0.2, Pr = 6.2, \lambda = -10$ and $\phi i = 0.05$.

**Figure 8:**
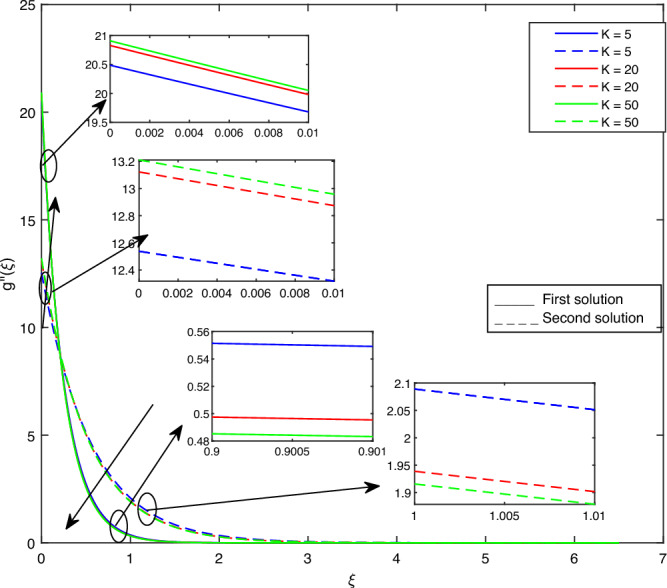
Portrays the existence of dual solutions on $g''(\xi )$ with variation of *K* when $M= 0.9, Ec = 1, \xi = 0.4, K= 50,Rd = 0.2, S= 5, \lambda 1 = 0.1, \lambda 2 = 0.2, \lambda 3 = 0.2, Pr = 6.2, \lambda = -10, \phi i = 0.05$ and $\phi j = 0.02$.

**Figure 9:**
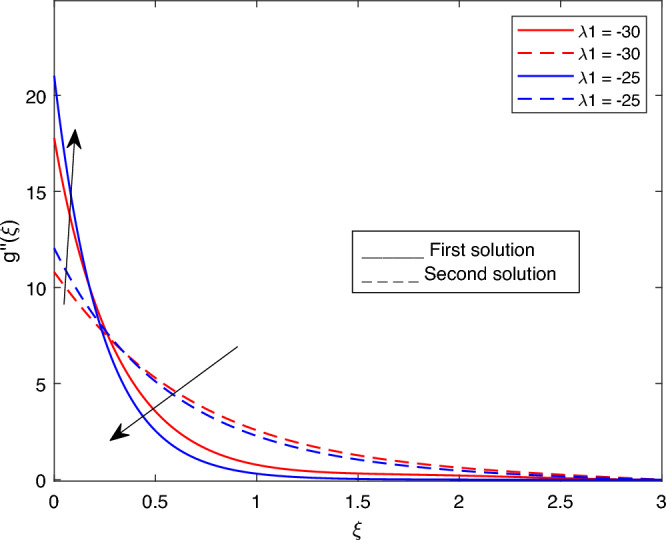
Portrays the existence of dual solutions on $g''(\xi )$ with variation of $\lambda 1$ when $M= 0.9, Ec = 1, \xi = 0.4, \beta = -1,Rd = 0.2, S= 5, \lambda 1 = 0.1, \lambda 2 = 0.2, \lambda 3 = 0.2, Pr = 6.2, \lambda = -10, \phi i = 0.05$ and $\phi j = 0.02$.

**Figure 10:**
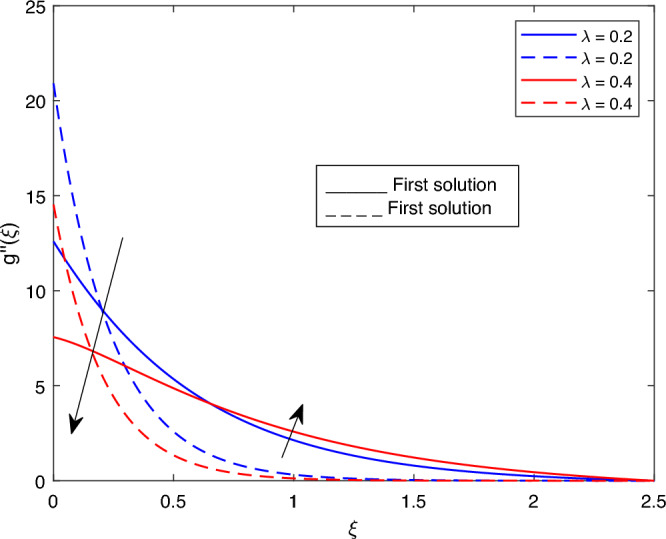
Portrays the existence of dual solutions on $g''(\xi )$ with variation of $\lambda 2$ when $M= 0.9, Ec = 1, \xi = 0.4, \beta = -1, K= 50, Rd = 0.2, S= 5, \lambda 2 = 0.2, \lambda 3 = 0.2, Pr = 6.2, \lambda = -10, \phi i = 0.05$ and $\phi j = 0.02$.

**Figure 11:**
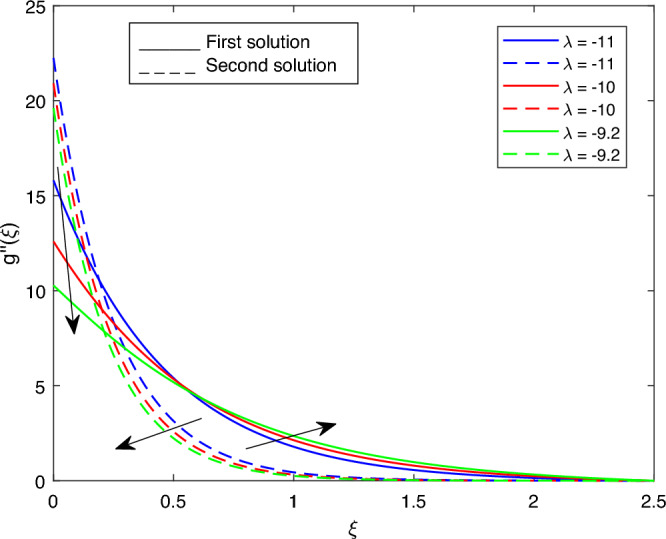
Portrays the existence of dual solutions on $g''(\xi )$ with variation of $\lambda$ when $M= 0.9, Ec = 1, \xi = 0.4, \beta = -1, K= 50,Rd = 0.2, S= 5, \lambda 1 = 0.1, \lambda 3 = 0.2, Pr = 6.2, \lambda = -10, \phi i = 0.05$ and $\phi j = 0.02$.

**Figure 12:**
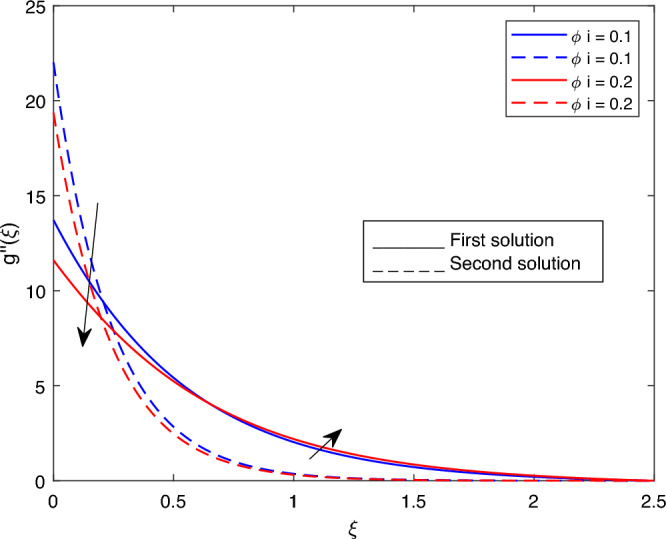
Portrays the existence of dual solutions on $g''(\xi )$ with variation of $\phi i$ when $M= 0.9, Ec = 1, \xi = 0.4, \beta = -1, K= 50,Rd = 0.2, S= 5, \lambda 1 = 0.1, \lambda 2 = 0.2, \lambda 3 = 0.2, Pr = 6.2$ and $\phi j = 0.02$.

**Figure 13:**
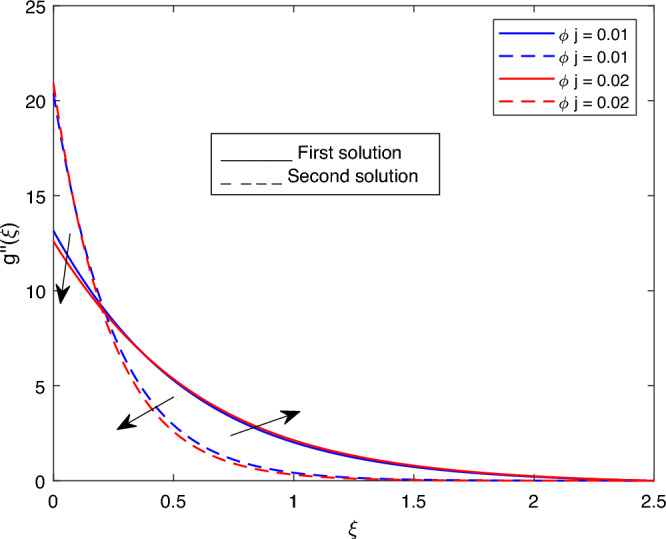
Portrays the existence of dual solutions on $g''(\xi )$ with variation of $\phi j$ when $M= 0.9, Ec = 1, \xi = 0.4, \beta = -1, K= 50,Rd = 0.2, S= 5, \lambda 1 = 0.1, \lambda 2 = 0.2, \lambda 3 = 0.2, Pr = 6.2, \lambda = -10$ and $\phi i = 0.05$.

**Figure 14:**
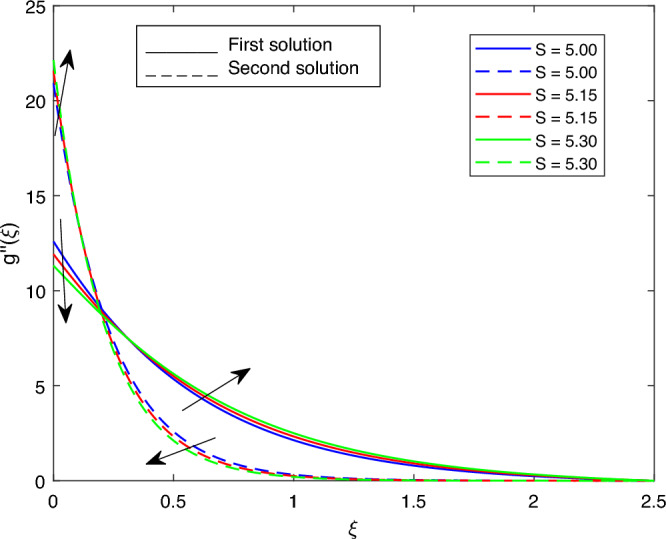
Portrays the existence of dual solutions on $g''(\xi )$ with variation of *S* when $M= 0.9, Ec = 1, \xi = 0.4, \beta = -1, K= 50,Rd = 0.2, \lambda 1 = 0.1, \lambda 2 = 0.2, \lambda 3 = 0.2, Pr = 6.2, \lambda = -10, \phi i = 0.05$ and $\phi j = 0.02$.

**Figure 15:**
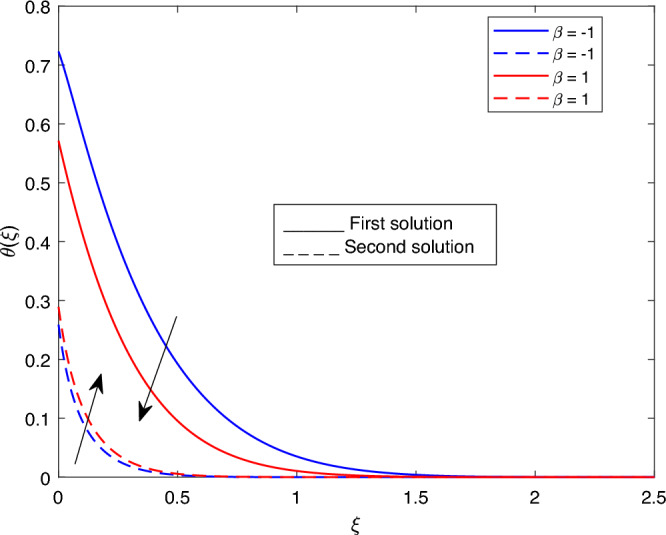
Portrays the existence of dual solutions on $\theta (\xi )$ with variation of $\beta$ when $M= 0.9, Ec = 1, \xi = 0.4, K= 50,Rd = 0.2, S= 5, \lambda 1 = 0.1, \lambda 2 = 0.2, \lambda 3 = 0.2, Pr = 6.2, \lambda = -10, \phi i = 0.05$ and $\phi j = 0.02$.

**Figure 16:**
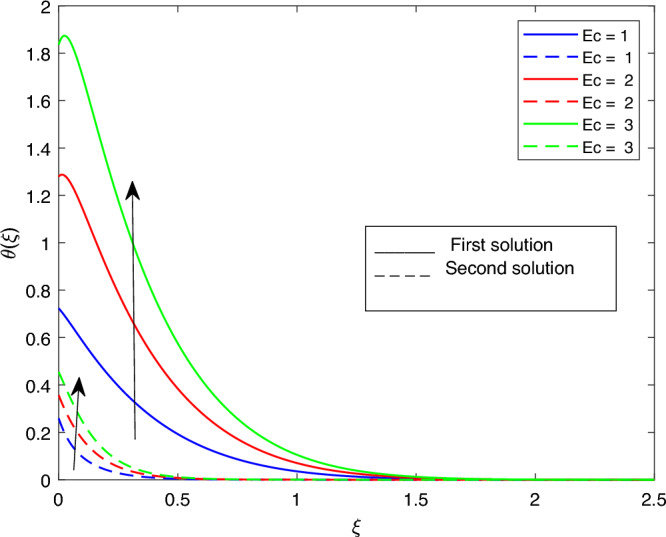
Portrays the existence of dual solutions on $\theta (\xi )$ with variation of *Ec* when $M= 0.9, \xi = 0.4, \beta = -1, K= 50,Rd = 0.2, S= 5, \lambda 1 = 0.1, \lambda 2 = 0.2, \lambda 3 = 0.2, Pr = 6.2, \lambda = -10, \phi i = 0.05$ and $\phi j = 0.02$.

**Figure 17:**
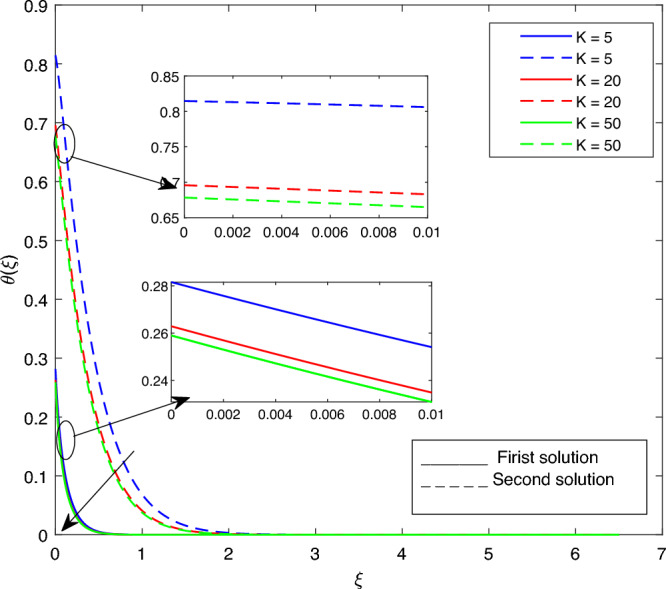
Portrays the existence of dual solutions on $\theta (\xi )$ with variation of *K* when $M= 0.9, Ec = 1, \xi = 0.4, \beta = -1, K= 50,Rd = 0.2, S= 5, \lambda 1 = 0.1, \lambda 2 = 0.2, \lambda 3 = 0.2, Pr = 6.2, \lambda = -10, \phi i = 0.05$ and $\phi j = 0.02$.

**Figure 18:**
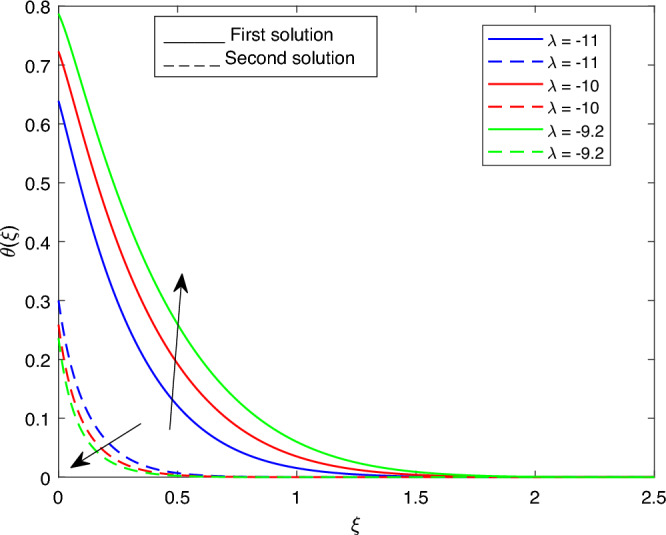
Portrays the existence of dual solutions on $\theta (\xi )$ with variation of $\lambda$ when $M= 0.9, Ec = 1, \xi = 0.4, \beta = -1, K= 50,Rd = 0.2, S= 5, \lambda 1 = 0.1, \lambda 2 = 0.2, \lambda 3 = 0.2, Pr = 6.2,\phi i = 0.05$ and $\phi j = 0.02$.

**Figure 19:**
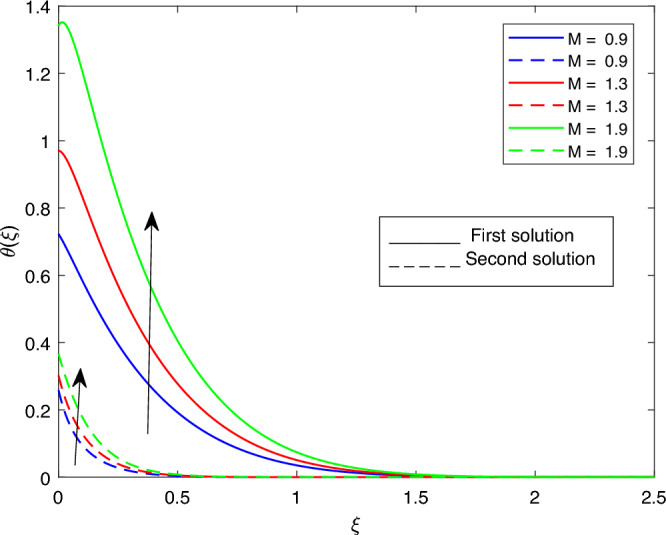
Portrays the existence of dual solutions on $\theta (\xi )$ with variation of *M* when $Ec = 1, \xi = 0.4, \beta = -1, K= 50,Rd = 0.2, S= 5, \lambda 1 = 0.1, \lambda 2 = 0.2, \lambda 3 = 0.2, Pr = 6.2, \lambda = -10, \phi i = 0.05$ and $\phi j = 0.02$.

**Figure 20:**
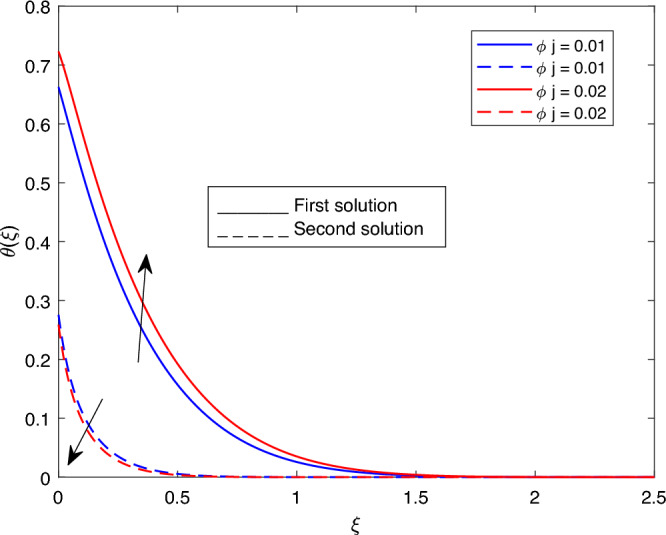
Portrays the existence of dual solutions on $\theta (\xi )$ with variation of $\phi j$ when $M= 0.9, Ec = 1, \xi = 0.4, \beta = -1, K= 50,Rd = 0.2, S= 5, \lambda 1 = 0.1, \lambda 2 = 0.2, \lambda 3 = 0.2, Pr = 6.2, \lambda = -10$ and $, \phi i = 0.05$.

**Figure 21:**
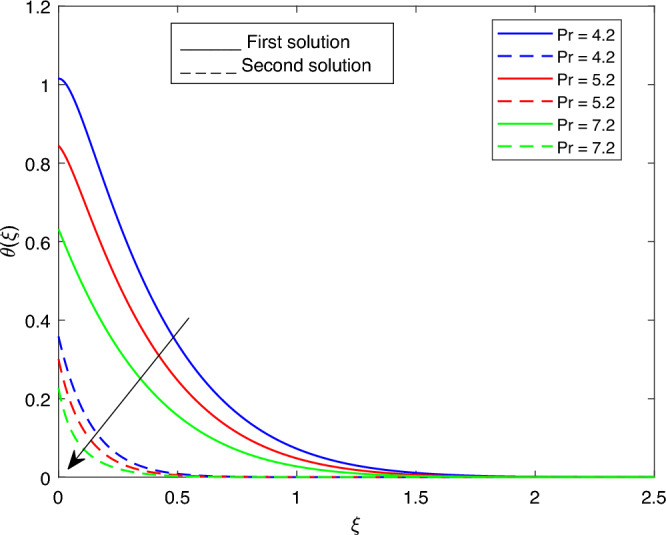
Portrays the existence of dual solutions on $\theta (\xi )$ with variation of *Pr* when $M= 0.9, Ec = 1, \xi = 0.4, \beta = -1, K= 50,Rd = 0.2, S= 5, \lambda 1 = 0.1, \lambda 2 = 0.2, \lambda 3 = 0.2, \lambda = -10, \phi i = 0.05$ and $\phi j = 0.02$.

**Figure 22:**
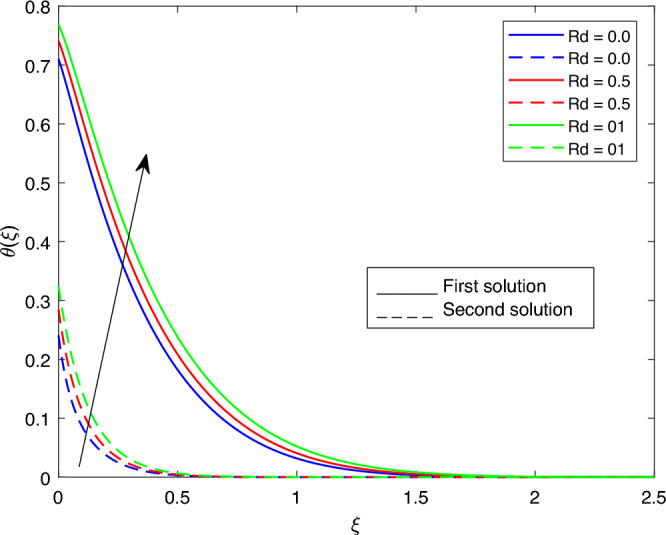
Portrays the existence of dual solutions on $\theta (\xi )$ with variation of *Rd* when $M= 0.9, Ec = 1, \xi = 0.4, \beta = -1, K= 50, S= 5, \lambda 1 = 0.1, \lambda 2 = 0.2, \lambda 3 = 0.2, Pr = 6.2, \lambda = -10, \phi i = 0.05$ and $\phi j = 0.02$.

**Figure 23:**
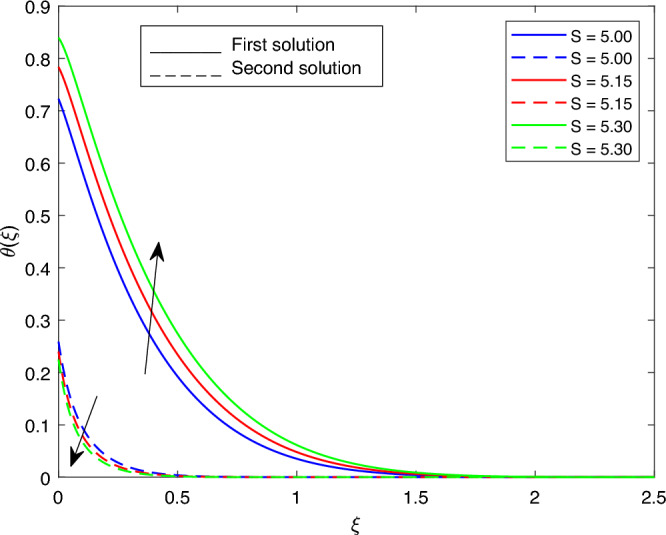
Portrays the existence of dual solutions on $\theta (\xi )$ with variation of *S* when $M= 0.9, Ec = 1, \xi = 0.4, \beta = -1, K= 50,Rd = 0.2, \lambda 1 = 0.1, \lambda 2 = 0.2, \lambda 3 = 0.2, Pr = 6.2, \lambda = -10, \phi i = 0.05$ and $\phi j = 0.02$.

**Figure 24:**
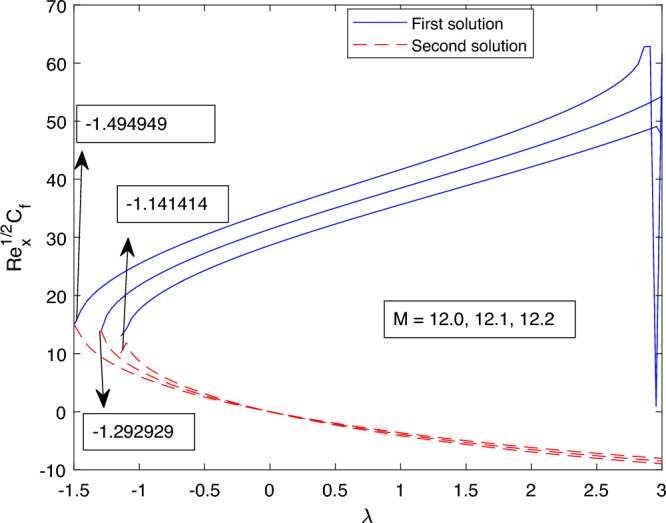
Portrays the existence of dual solutions on $Re_s^{1/2}C_f$ with variation of *M*.

**Figure 25:**
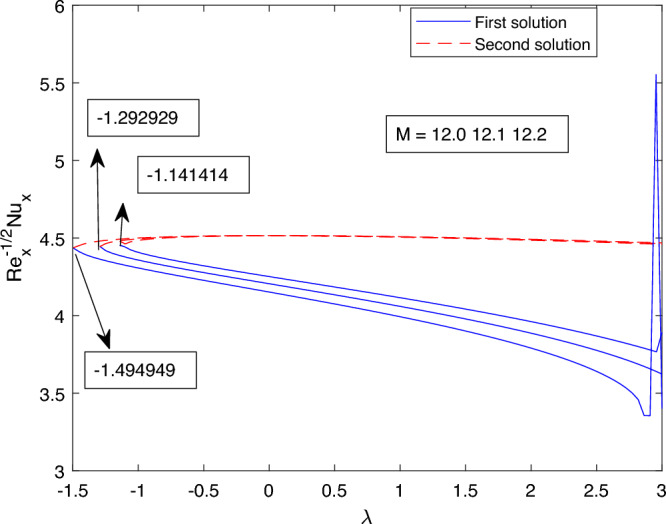
Portrays the existence of dual solutions on $Re_s^{-1/2}Nu_s$ with variation of *M*.

Figures 2, 3, 4, 5 and Fig. 6 depict the velocity profile $g'(\xi )$ with variations of $\lambda$, *S*, $\lambda 1$, and $\lambda 2$. Figures 2 and 3 illustrate the velocity profile $g'(\xi )$ with variations of $\lambda$ and *S*, respectively. In these figures, boundary layer thickness of the first solution is greater than the second solution. As the values of both $\lambda$ and *S* increase, the velocity profile for the first solution decreases and the opposite trend is observed for the second solution. Figure 4 portrays the velocity profile $g'(\xi )$ with a variation of $\lambda 1$. In this figure, the boundary layer thickness of the first solution is smaller than that of second solution. Physically, this is due to the fact that an enhancement in $\lambda 1$ increases the buoyancy force, which results in an increase in the velocity profile for both solutions. Figure 5 labels the velocity profile $g'(\xi )$ with a variation of $\lambda 2$. In this figure, the boundary layer thickness of the first solution is grater than the second solution. It is physically noted that the velocity profile for first solution is increasing when $\lambda 2$ increases. As a result, the opposite trend was observed for both solutions when various values of $\lambda 2$ were presented. Figure 6 portrays the velocity profile $g'(\xi )$ with variation of $\phi j$.In this figure, the boundary layer thickness of the first solution is greater than the second solution. As the values of $\phi j$ increase, the velocity profile for the second solution decreases, and the opposite trend is observed for the first solution.

Figures 7, 8, 9, 10, 11, 12, 13 and 14 depict the shear stress profile $g''(\xi )$ with variations of $\beta$, *K*, $\lambda 1$, $\lambda 2$, $\lambda$, and *S*. Figure 7 illustrates the shear stress profile $g''(\xi )$ with a variation of $\beta$. In this figure, the boundary layer thickness of first solution is greater than the second solution. It can be observed that the thickness of the shear stress increases or decreases for the second or first solution. As a result, the shear stress profile increased for the second solution, and the opposite trend was observed for the first solution. Figures 8 and 9 delineate the shear stress profile $g''(\xi )$ with variations of *K* and $\lambda 1$, respectively. It can be observed from both figures that the thickness of the shear stress profile for the first solution is larger than that of the second solution, even though after some interval point of $\xi$ the opposite trend was observed. As a result, the shear stress profile increased for the first solution, and the opposite trend was observed for the second solution, though after some interval, the inverse of this statement was observed. Figures 10, 11 and 12 label the shear stress profile $g''(\xi )$ with variations of $\lambda 2$,$\lambda$ and $\phi i$. It can be observed that from all Figs. 10, 11 and 12 the shear stress profile increased for the first solution at some interval point of $\xi$, and after this interval, inverse of this statement was observed. Even though the shear stress profile decreases for the second solution in all Figs. 10, 11 and 12. Figures 13 and 14 illustrate the shear stress profile $g''(\xi )$ with variation of the volume fraction of $Al_{2}O_{3}$ $\phi j$ and mass suction parameter *S*. From Fig. 13 and 14, we came to understand that in both figures, the thickness of the shear stress profile for first solution is smaller than second solution even though after some interval point of $\xi$ the opposite trend was observed. As a result, the shear stress profile decrease for the first solution , and the opposite trend was observed for the second solution, even though some interval points of $\xi$ inverse of this statement were observed.

Figures 15, 16, 17, 18, 19, 20, 21, 22 and 23 show the effects of the temperature profile $\theta (\xi )$ with various values of $\beta$, *Ec*, *K*, $\lambda$, *M*, $\phi j$, *Pr*, *Rd*, and *S*. Figure 15 sketches the temperature profile $\theta (\xi )$ with a variation of $\beta$. It is observed from this figure that the thickness of the thermal boundary layer for the second solution is smaller than the first solution. As the values of $\beta$ increase, the temperature profile for the first solution increases and the opposite trend is observed for the second solution. Figure 16 sketches the temperature profile $\theta (\xi )$ with variation of *Ec*. It is observed from this figure that the thickness of the thermal boundary layer for the second solution is smaller than the first solution. Physically,the ratio of the dynamic temperature to the temperature or the kinetic energy to the enthalpy driving force for heat transfer is *Ec*. As a result, as the values of *Ec* increase, the temperature profiles for both solutions increase. Figure 17 sketches the temperature profile $\theta (\xi )$ with a variation of *K*. The thickness of the thermal boundary layer becomes thinner as the *K* impact is increased, even though for the second solution is larger than the first solution. As the values of *K* increase the temperature profile of both the first and second solutions decreases. Figure 18 depicts the temperature profile $\theta (\xi )$ with a variation of $\lambda$. It is seen from this figure that the thickness of the thermal boundary layer for the second solution is smaller than the first solution. As a result, The second solution is a decrease; the opposite trend was observed. Figure 19 sketches the temperature profile $\theta (\xi )$ with variations of *M*. It is seen from this figure that the thermal boundary layer thickness for the first solution is greater than that of the second solution. Physically, the Lorentz force due to the transverse magnetic field has the property of relaxing the fluid velocity and temperature distributions. Accordingly, the temperature distribution of both solutions enlarges as the values of the magnetic parameter *M* increase. Figure 20 sketches the temperature profile $\theta (\xi )$ with variation of $\phi j$. It can be observed that the thermal boundary layer thickness increases or decreases for the second or first solution. As a result, the temperature profile increased for the first solution, and the opposite trend was observed for the second solution via $\phi j$. Figure 21 sketches the temperature profile $\theta (\xi )$ with variation of *Pr*. It is seen from this figure that the thermal boundary layer thickness for the first solution is larger than the second solution. physically, the ratio of momentum diffusivity to thermal diffusivity or kinematic viscosity to thermal diffusivity. From this fact, for various values of the *Pr,* temperature profile have opposite characteristics for both solutions. Figure 22 delineates the temperature profile $\theta (\xi )$ with variation of *Rd*. The fact that the relative contribution of conduction heat transfer to thermal radiation transfer is a radiation parameter. As it can be seen from the figure, the thermal boundary layer thickness for the first solution is larger than that of the second solution. This shows that as the rate of radiation increases, the temperature profile in both solutions also increases. Figure 23 sketches the temperature profile $\theta (\xi )$ with variation of *S*. It is seen from this figure that the thermal boundary layer thickness for first solution is greater than that of the second solution. As a result, for both solutions, the opposite trend in the suction parameter is increased.

The impact of the dual solution and the critical value for various values of *M* on the skin friction coefficient and the local Nusselt number are portrayed in Figs. 24 and 25 respectively. Figure 24 portrays the behavior of the dual solution and the critical values for M on the skin friction coefficient. Critical value for M = 12.0, 12.1, and 12.2 are $\lambda c1 = -1.141414, \lambda c2 = -1.292929$, and $\lambda c3 = -1.494949$ respectively, where $\lambda < 0$ up to which the solution exists. Dual solution exist between $\lambda ci < \lambda$ where i = 1,2 and 3. For the value of *M* uphill, of the absolute value of $\lambda ci$ increase for both the skin friction coefficient and the local Nusselt number. The same property, which can be easily understood is registered in Fig. 25, delineates the characteristic of dual solution and critical value for various value of *M* on the local Nusselt number.

Table 2 summarizes the current result and compares it with previous work in the open literature for various values of *K* on -*Cfs*. The result confirms good agreement, this shows that the present method gives an acceptable result. Tables 3 and 4 show the effect of dual solutions with varying *M*, *S*, *Br*, and *Pr* values on the $g''(0)$, *Cfs*, and *Nus* of $Cu-Al_{2}O_{3}/H_{2}O$, a hybrid nano-fluid. The results present the properties of the non-dimensional parameters *M*, *S*, *Br*, and *Pr* on $g''(0)$, *Cfs*, and *Nus* in an easy-to-understand manner, as shown in Tables 3 and 4. For different estimations of *Pr* the smallest eigenvalue are shown in Table 5. From this Table 5 we generalize that the first solution is stable (physically realizable) whereas the second solution is unstable (not physically realizable).

**Table 5:** portrays the smallest eigenvalues $\gamma$ at several values of *Pr* when $Ec = 1, \xi = 7, \beta = -2, K= 2.5, Rd= 0.2, S= 2.5, \lambda 1 = 0.1, \lambda 2 = 0.3, \lambda 3 = 0.2, M = 0.9, \lambda = -9, \phi i = 0.1$ and $\phi j = 0.1$.

*Pr*	Smallest eigenvalues $\gamma$	
	First solution	Second solution
10.2	0.0165	− 0.1021
9.2	0.0151	− 0.1020
7.2	0.0117	− 0.1019
6.2	0.0095	− 0.1018
5.2	0.0071	− 0.1017
3.2	0.0017	− 0.1016

## Conclusions

In this study, two-dimensional, incompressible, viscous boundary layer flow of hybrid nanofluid with stability analysis and a dual solution of mixed convection and thermal radiation with hybrid nano-fluid over curved surfaces past a stretching/shrinking surface are considered under slip conditions. A hybrid nanofluid,in which water is used as the base fluid and copper and alumina are used as nano-particles, and magnetic field are taken into account. The bvp4c method is used to solve the numerical solution of the governing equations of the present study. The main outputs of this study are:

- For the values of both $\lambda$ and *S*, the velocity profile for the first solution decreased, and the opposite trend was observed for the second solution.
- As the values of $\phi j$ increase, the velocity profile for the second solution decreases, and the opposite trend is observed for the first solution.
- As the values of $\beta$ increase, the shear stress profile increases for the second solution, and the opposite trend is observed for the first solution.
- For the values of *K* and $\lambda 1$, the shear stress profile increased for the first solution, and the opposite trend was observed for the second solution, though after some interval points, the inverse of this statement was observed.
- For the values of *K*, the temperature profiles increase, and for both the first and second solutions, they decrease.
- As the values of *Ec* increase, the temperature profiles for both solutions increase.
- For the values of $S,Pr,Rd,M, \;\text{and}\; \lambda$, the upwind thermal boundary layer of the first solution is larger than the second solution.
- For the value of *M* uphill, the estimation of the absolute value of $\lambda ci$ increases for both the skin friction coefficient and the local Nusselt number.
- In the second solution, increasing the values of $\beta$, *Pr*, *S*, *Ec*, and *M* has a similar effect on $g''(0)$, *Cfs*, -$\theta '(0)$,and *Nus*.
- In the first solution, increasing the values of *Ec*, *S*, and *Pr* on $g''(0)$ and *Cfs* result in a decrease.
- The first solution has a positive eigenvalue, whereas the second solution has a negative eigenvalue.

## Data Availability

All data generated or analysed during this study are included in this published article.

## List of symbols

*M*: Hartman number (−)
*Pr*: Prandtl number (−)
$\beta$: Parameter of unsteadiness (−)
*K*: Curvature parameter (−)
$\lambda 1$: Mixed convection parameterv
*Gr*: Local Grashof number
*Rd*: Radiation parameter (−)
*Ec*: Eckert number (−)
$g'(\xi )$: Dimensionless velocity function (−)
*Nus*: Local Nusselt number (−)
*Res*: Local Reynolds number (−)
*Cfs*: Local skin friction coefficient (−)
*S*: Mass suction parameter (−)
$\lambda 3$: Thermal jump parameter (−)
$\lambda 2$: Velocity slip parameter (−)
$\lambda$: Shrinking/stretching parameter (−)
$\phi$: Nano-particle volume fraction (−)
*t*: Time coordinate (s)
*T*: Temperature field ($$\theta$$)
$$T_{\infty }$$: Ambient temperature ($$\theta$$)
$$T_{w}$$: Reference temperature ($$\theta$$)
*R*: Radius of curvature (m)
*u*, *v*: Velocity components (m/s)
*s*, *r*: Spatial coordinates (m)
*a*: Constant (−)
$\sigma ^{*}$: Stefan–Boltzmann constant (−)
$K^{*}$: Coefficient of mean absorption −
$\theta (\xi )$: Dimensionless temperature function (−)
$k _{hnf}$: Thermal conductivity of hybrid nanofluid ($\hbox {Wm}^{-1}\;\hbox {K}^{-1}$)
$\beta _{hnf}$: Thermal expansion coefficient of hybrid nanofluid ($\hbox {K}^{-1}$)
$\xi$: Dimensionless scale in radial direction (−)
$\sigma _{hnf}$: Electrical conductivity of hybrid nanofluid (S/m)
$\mu _{hnf}$: Dynamic viscosity of hybrid nanofluid ($\hbox {kgm}^{-1}\;\hbox {s}^{-1}$)
$Cp _{hnf}$: Heat capacity of hybrid nanofluid ($\hbox {J}/(\hbox {kg}\;\hbox {K}))$)
$\rho _{hnf}$: Density of the hybrid nanofluid ($\hbox {kgm}^{-3}$)
$\sigma _{nf}$: Electrical conductivity of nanofluid (S/m)
$\mu _{nf}$: Dynamic viscosity of nanofluid ($\hbox {kgm}^{-1}\;\hbox {s}^{-1}$)
$Cp _{nf}$: Heat capacity of nanofluid ($\hbox {J}/(\hbox {kg}\;\hbox {K}))$)
$\rho _{nf}$: Density of the nanofluid ($\hbox {kgm}^{-3}$)
$k _{nf}$: Thermal conductivity of nanofluid ($\hbox {Wm}^{-1}\;\hbox {K}^{-1}$)
$\beta _{nf}$: Thermal expansion coefficient of nanofluid ($\hbox {K}^{-1}$)
$$\sigma$$: Electrical conductivity (S/m)
$\mu$: Dynamic viscosity of the fluid ($\hbox {kgm}^{-1}\;\hbox {s}^{-1}$)
*Cp*: Specific heat at constant pressure ($\hbox {J}\,\hbox {kg}^{-1}\;\hbox {K}^{-1}$)
$\rho$: Density of the fluid ($\hbox {kgm}^{-3}$)
*k*: Thermal conductivity of the fluid ($\hbox {Wm}^{-1}\;\hbox {K}^{-1}$)

## Subscripts

*hnf*: Hybrid nanofluid
*w*: Reference
$$\infty$$: Ambient
*nf*: Nanofluid
*f*: Fluid
*ni*: Cu
*nj*: Al$$_{2}$$O$$_{3}$$
